# Supplementary material for: Conservation and divergence of myelin proteome and oligodendrocyte transcriptome profiles between humans and mice
Source: eLife. 2022 May 11;11:e77019. doi: 10.7554/eLife.77019 (PMC9094742; doi:10.7554/eLife.77019)
Supplement: Figure 3—source data 1. [file elife-77019-fig3-data1.docx]

**Gargareta et al., Figure 3-source data 1**

*Parameters applied for scRNA-seq individual dataset quality control and integrative analysis*

| Publication | Cell Filter | PCs for Embedding/ PCs for Embedding Healthy OLG only (Resolution) | Original Cell Number (including none OLGs and/or non-healthy) | Recovered OPCs/NFOs/MOL |
| --- | --- | --- | --- | --- |
| MOUSE ALL | - | /20 (0.5) | 108,390 | 1,957/10,391/95,966 |
| Falcão et al. (2018)  GSE113973 | Cells from EAE mice were excluded  (see Methods Falcão et al. (2018)) | /14 (0.5) | 1,765 | 116/17/612 |
| Marques et al. (2016)  GSE775330 | (see Methods Marques et al. (2016)) | 20 (0.5) | 5,069 | 310/652/4,031 |
| Saunders et al. (2018)  GSE116470 | Cell types other than OLG were excluded  (see Methods Saunders et al. (2018)) | / | 49,270 | 0/0/49,270 |
| Wheeler et al. (2020)  GSE130119 | Cell types other than OLG were excluded;  Cells from EAE mice were excluded  (see Methods Wheeler et al. (2020)) | 30 (0.5) | 71,310 | 0/0/1,373 |
| Ximerakis et al. (2019)  GSE129788 | Cell types other than OLG were excluded;  Mt: <30%  (see Methods  Ximerakis et al.  (2019)) | 40 (0.5) / 13 (0.5) | 36,908 | 0/0/15,400 |
| Zeisel et al. (2018)  SRP135960 | Cell types other than OLG were excluded  (see Methods  Zeisel et al. (2018)) | / 20 (0.5) | 31,073 | 820/9,678/20,575 |
| Zeisel et al. (2015)  GSE60361 | Cell types other than OLG were excluded  (see Methods  Zeisel et al. (2015)) | 30 (0.5) / 22 (0.5) | 3,005 | 0/0/820 |
| Zhou et al. (2020)  GSE140511 | Cell types other than OLG were excluded;  No Cut-off | 40 (0.5)/ 7 (0.5) | 90,634 | 711/44/3,885 |
| HUMAN ALL | - | /15 (0.4) | 55,242 | 41517/132/3700 |
| Grubman et al. (2019)  GSE138852 | Cells from AD patients were excluded  Mt: <10%  (see Methods Grubman et al. (2019) ) | 20 (0.5) / 7 (0.5) | 13,214 | 899/0/2,777 |
| Habib et al. (2017)  Single Cell Portal DroNC-Seq human archived brain | > 10,000 reads  per nucleus  (see Methods Habib et al. (2017)) | 20 (0.5) / 30 (0.5) | 14,963 | 0/0/2,936 |
| Jäkel et al. (2019)  GSE118257 | Cells from MS patients were excluded  Mt: <20%  (see Methods Jäkel et al. (2019)) | 25 (0.5) / 10 (0.5) | 17,569 | 273/132/3,519 |
| Lake et al. (2018)  GSE97930 | Mt genes excluded  (see Methods  Lake et al. (2018)) | /10 (0.5) | 5,690 | 1,337/0/4,353 |
| Wheeler et al. (2020)  GSE130119 | Cell types other than OLG were excluded  Cells from MS patients were excluded  MT: < 30%  (see Methods Wheeler et al. (2020) | 20 (0.5) /20 (0.5) | 30,606 | 0/0/10,534 |
| Zhou et al. (2020)  syn21125841 | Cell types other than OLG were excluded  No Cut-off | /12 (0.5) | 33,694 | 1,191/0/17,398 |
| MERGE ALL (MOL only) | - | /20 (0.1) | 146,239 | 3,700/132/41,517 |
