# Supplementary material for: Conservation and divergence of myelin proteome and oligodendrocyte transcriptome profiles between humans and mice
Source: eLife. 2022 May 11;11:e77019. doi: 10.7554/eLife.77019 (PMC9094742; doi:10.7554/eLife.77019)
Supplement: Transparent reporting form [file elife-77019-transrepform1.pdf]

eLIFE
1st Floor
24 Hills Road
Cambridge CB2 1JP, UK
P 01223 855340
W elifesciences.org
T @elife

eLife's transparent reporting form

We encourage authors to provide detailed information *within their submission* to facilitate the interpretation and replication of experiments. Authors can upload supporting documentation to indicate the use of appropriate reporting guidelines for health-related research (see EQUATOR Network), life science research (see the BioSharing Information Resource), or the ARRIVE guidelines for reporting work involving animal research. Where applicable, authors should refer to any relevant reporting standards documents in this form.

If you have any questions, please consult our Journal Policies and/or contact us: editorial@elifesciences.org.

Sample-size estimation

- You should state whether an appropriate sample size was computed when the study was being designed
- You should state the statistical method of sample size computation and any required assumptions
- If no explicit power analysis was used, you should describe how you decided what sample (replicate) size (number) to use

Please outline where this information can be found within the submission (e.g., sections or figure legends), or explain why this information doesn't apply to your submission:

Explicit power analysis was not performed. Sample sizes were decided according toprior experience with analyzing myelin using similar methods as in the present work.

Replicates

- You should report how often each experiment was performed
- You should include a definition of biological versus technical replication
- The data obtained should be provided and sufficient information should be provided to indicate the number of independent biological and/or technical replicates
- If you encountered any outliers, you should describe how these were handled
- Criteria for exclusion/inclusion of data should be clearly stated
- High-throughput sequence data should be uploaded before submission, with a private link for reviewers provided (these are available from both GEO and ArrayExpress)

Please outline where this information can be found within the submission (e.g., sections or figure legends), or explain why this information doesn't apply to your submission:

For the proteome analysis of human myelin, five biological replicates were analyzed, with each biological replicate analyzed as two technical replicates. In Immunoblotting analysis each lane represents one biological replicate. In Immunohistochemistry, three biological replicates were analyzed, and one representative biological replicate is depicted. Each biological replicate equals a sample from one individual person, as specified in the methods section. The technical replicates apply only to the proteome experiments. More details about the biological and technical replicates are given in the respective method section, and for the proteome dataset additionally in Figure 1-source data 1.

Statistical reporting

- Statistical analysis methods should be described and justified
- Raw data should be presented in figures whenever informative to do so (typically when N per group is less than 10)
- For each experiment, you should identify the statistical tests used, exact values of N, definitions of center, methods of multiple test correction, and dispersion and precision measures (e.g., mean, median, SD, SEM, confidence intervals; and, for the major substantive results, a measure of effect size (e.g., Pearson's r, Cohen's d)
- Report exact p-values wherever possible alongside the summary statistics and 95% confidence intervals. These should be reported for all key questions and not only when the p-value is less than 0.05.

Please outline where this information can be found within the submission (e.g., sections or figure legends), or explain why this information doesn't apply to your submission:

Statistical information is given in the Methods section "Statistics and reproducibility". For thescatter plots, Pearson correlation and regression line, values (r) are respectively given in Figure 1, Figure 1-figure supplement 2 and Figure 2. The scRNA-seq cluster marker analysis was conducted using MAST algorithm, the marker genes are given in Figure 4-source data 1.

(For large datasets, or papers with a very large number of statistical tests, you may upload a single table file with tests, Ns, etc., with reference to sections in the manuscript.)

Group allocation

- Indicate how samples were allocated into experimental groups (in the case of clinical studies, please specify allocation to treatment method); if randomization was used, please also state if restricted randomization was applied
- Indicate if masking was used during group allocation, data collection and/or data analysis

Please outline where this information can be found within the submission (e.g., sections or figure legends), or explain why this information doesn't apply to your submission:

Myelin purification and proteome analysis of human myelinincluded five post-mortem subjects. Both males and females were included, ranging between 55 to 75 years of age as specified in the method section "Human samples".

Additional data files ("source data")

- We encourage you to upload relevant additional data files, such as numerical data that are represented as a graph in a figure, or as a summary table
- Where provided, these should be in the most useful format, and they can be uploaded as "Source data" files linked to a main figure or table
- Include model definition files including the full list of parameters used
- Include code used for data analysis (e.g., R, MatLab)
- Avoid stating that data files are "available upon request"

Please indicate the figures or tables for which source data files have been provided:

Figure 1-source data 1: Identification and label-free quantification of proteins in human CNS myelin and white matter homogenate bytwo different acquisition modes
Figure 2-source data 1: Labeled original immunoblots
Figure 3-source data 1: Parameters for scRNA-seq individual dataset quality control and integrativeanalysis
Figure 4-source data 1: MAST calculated marker genes from human and mouse integrated MOL subpopulations
